# Modelling physical resilience in ageing mice

**DOI:** 10.1016/j.mad.2018.10.001

**Published:** 2018-10-02

**Authors:** Markus Schosserer, Gareth Banks, Soner Dogan, Peter Dungel, Adelaide Fernandes, Darja Marolt Presen, Ander Matheu, Marcin Osuchowski, Paul Potter, Coral Sanfeliu, Bilge Guvenc Tuna, Isabel Varela-Nieto, Ilaria Bellantuono

**Affiliations:** a University of Natural Resources and Life Sciences, Vienna, Department of Biotechnology, Vienna, Austria; b Mammalian Genetics Unit, MRC Harwell Institute, Harwell Campus, Oxfordshire, OX11 0RD, United Kingdom; c Department of Medical Biology, School of Medicine, Yeditepe University, Istanbul, Turkey; d Ludwig Boltzmann Institute for Experimental and Clinical Traumatology, AUVA Research Center, Vienna, Austria; e Neuron-Glia Biology in Health and Disease, iMed.ULisboa, Research Institute for Medicines, Department of Biochemistry and Human Biology, Faculty of Pharmacy, Universidade de Lisboa, Lisboa, Portugal; f Oncology Department, Biodonostia Research Institute, San Sebastián, Spain; g Institute of Biomedical Research of Barcelona (IIBB) CSIC, IDIBAPS, CIBERESP, Barcelona, Spain; h Department of Medical Biophysics, School of Medicine, Yeditepe University, Istanbul, Turkey; i "Alberto Sols" Biomedical Research Institute CSIC-UAM, Madrid, Spain; j MRC/Arthritis Research-UK Centre for Integrated Research into Musculoskeletal Ageing (CIMA), Department of Oncology and Metabolism, The Medical School, Beech Hill Road, Sheffield, S10 2RX, United Kingdom

**Keywords:** Ageing, Physical resilience, Mouse models, Frailty, Stress recovery, Intervention testing

## Abstract

Geroprotectors, a class of drugs targeting multiple deficits occurring with age, necessitate the development of new animal models to test their efficacy. The COST Action MouseAGE is a European network whose aim is to reach consensus on the translational path required for geroprotectors, interventions targeting the biology of ageing. In our previous work we identified frailty and loss of resilience as a potential target for geroprotectors. Frailty is the result of an accumulation of deficits, which occurs with age and reduces the ability to respond to adverse events (physical resilience). Modelling frailty and physical resilience in mice is challenging for many reasons. There is no consensus on the precise definition of frailty and resilience in patients or on how best to measure it. This makes it difficult to evaluate available mouse models. In addition, the characterization of those models is poor. Here we review potential models of physical resilience, focusing on those where there is some evidence that the administration of acute stressors requires integrative responses involving multiple tissues and where aged mice showed a delayed recovery or a worse outcome then young mice in response to the stressor. These models include sepsis, trauma, drug- and radiation exposure, kidney and brain ischemia, exposure to noise, heat and cold shock.

## 1 Introduction

Due to significant improvements in medical care in the past century, life expectancy in the western world is steadily increasing. The fastest growing segment of the population is people aged over 80 years (European Commission, 2009). Up to half of these individuals have frailty, which is defined as an accumulation of deficits, and is often associated with multiple age-related diseases and loss of resilience, i.e., the ability to resist or recover from adverse events (Hadley et al., 2017; LeBrasseur, 2017; Whitson et al., 2016). Triggers can include challenges, which have considerable toxicity such as chemotherapy, or an event such as an infection or fall

Recent progress in the field of ageing research has led to the development of interventions, which have the potential to target ageing-associated diseases and frailty, also termed “geroprotectors” (Figueira et al., 2016). These drugs target the biology of ageing and their main characteristic is the ability to target more than one deficit at the same time (Bellantuono, 2018). For this reason, they are believed to have the potential to improve physical resilience and response to adverse events in frail older individuals. To support the regulatory approval of a clinical trial, appropriate testing in animal models is usually required. Mouse models are often the species of choice because they have many features similar to humans, they are small in size (meaning they require less drug during testing, reducing costs), and there is a vast body of knowledge around their use. However, modelling frailty in mice is challenging. Methods to assess frailty, such as the clinical frailty index (Whitehead et al., 2014), have recently been introduced in order to provide researchers with a platform to assess frailty, and an increase in the frailty index has been reported with age in C57BL/6 mice. However, the increase occurs over a long period of time and is modest.

Since reduced physical resilience is a feature of frailty with an important clinical impact, we and others propose the evaluation of novel geroprotectors using mouse models of physical resilience where mice are challenged with stressors to the point of showing a poor restorative response without the intervention (Kirkland et al., 2016). We support the statement of The American Geriatric Society Interventions on Frailty Working Group that has recognized that “a physically frail state may be clinically detected before disability, as well as a more advanced state of ‘clinically overt’ physical frailty that has already determined some initial degree of functional disability” (Ferrucci et al., 2004). For this reason we argue that physical resilience should be tested in ageing mice, when physiological reserves are diminished but the mice do not show severe functional disability yet. The reasoning is that mechanisms considered to be ‘hallmarks of ageing’ are dysfunctional (López-Otín et al., 2013) and thought to contribute to and possibly underpin the loss of resilience. For example, dysfunction of DNA repair pathways with age weakens the ability to recover from the administration of DNA damaging agents such as chemo- and radiotherapy. Impaired proteostasis or increased mitochondrial damage will affect the response to temperature stress, while fewer functional stem- and progenitor cells will impair tissue regeneration after trauma or ischemic events. Importantly, many of these stress-response pathways are part of a complex integrative regulatory network that is fully functional and has good physiological reserve in the young, allowing them to respond effectively to the stressor, while ageing mice will show decreased resilience. In our view, challenging ageing mice after administration of geroprotectors matches the clinical situation more closely than challenging young mice even if using harsher stressors as suggested by others (Hadley et al., 2017; Kirkland et al., 2016; LeBrasseur, 2017).

For this aim, appropriate physical challenges, as well as outcome measurements need to be carefully selected (LeBrasseur, 2017). Whenever technically feasible, we propose to perform dose-response and time-course measurements during stress- and recovery periods, to determine whether an intervention increases resistance, recovery or both.

Some environmental stressors that can be applied to mice are similar to those which may affect a frail person and from which they may not recover such as infection, trauma, ischemic events in the kidney and brain, as well as the use of medicinal drugs such as chemotherapy and radiation during cancer therapy.

In this review, we consider acute stressors, which require integrative responses involving multiple tissues and organs. For this we have looked for measurement of performance across the organ systems including cognitive, musculoskeletal, cardiovascular, metabolic, and immune systems. We have looked for evidence that aged mice showed a delayed recovery or a worse outcome than young mice in response to the stressor (Table 1). We have steered away from models which apply multiple tissue-specific challenges to obtain such responses to avoid adaptation to stress (hormesis), which may act as confounding factor (Calabrese et al., 2007). We have also not reviewed models which were described by Kirkland et al., (2016) and for which no additional knowledge was available at the time of writing of this review, such as starvation, de-hydration and physical exhaustion. Since we decided to focus the present review solely on physical resilience, we excluded psychological stressors, such as isolated housing or induction of fear and depression. We recommend reading this review in combination with that of (Kirkland et al., 2016).

## 2 Sepsis and endotoxin challenge

Older people have a higher susceptibility to infection and sepsis and associated increase in hospital mortality than younger adults (Ginde et al., 2013). Older patients are prone to develop delirium, even after apparently innocuous infection (van Gool et al., 2010). In addition, survivors of sepsis show cognitive impairment and functional disability (Iwashyna et al., 2010).

The most commonly used mouse models include endotoxin/LPS injection, as well as induction of sepsis by monobacterial induced pneumonias (e.g. *S. pneumoniae*, *P. aeruginosa*) (Coopersmith et al., 2002), and intra-abdominal infection produced by a single injection of faecal slurry (Secor et al., 2010; Tyml et al., 2017) or by caecal ligation and puncture (CLP). The latter constitutes a surgical intervention, whose severity can be modulated by the length of the caecum that is ligated and/or the diameter of the needle puncture through which the faecal mass leaks (Dejager et al., 2011).

Aged mice have been shown to be sensitive to a 6.5-fold lower lethally toxic dose of lipopolysaccharide (LPS) than young mice, and to have a quicker and more pronounced cytokine response when exposed to lower non-lethal doses (Saito et al., 2003; Tateda et al., 1996). Apart from changes in inflammatory markers, aged animals exposed to LPS also showed signs of multiple-tissue damage. For example, they displayed increased number, size, and total area of LPS-induced brain microhemorrhages, associated with increased blood-brain barrier disruption (BBB) and glia activation (Sumbria et al., 2018), higher cardiotoxicity, acute renal failure (Miyaji et al., 2003) and abnormal lipid metabolism with increased lipid accumulation in the liver (Chung et al., 2015). In addition, in an experimental mouse model of neurodegenerative disease, transient systemic inflammation was associated with acute exacerbation of cognitive and motor impairments and rapid disease progression (Cunningham and Hennessy, 2015). Peripheral sepsis following CLP also led to cognitive deficits in sepsis-survivor rats (Schwalm et al., 2014) and mice (Wu et al., 2015), which was associated with brain inflammatory response, increased amyloid β deposition, and decreased expression of synapse markers.

Interventions, known to delay ageing were administered in aged animals following sepsis. Voluntary exercise led to a better outcome in male 22-month non-obese C57BL/6 J mice induced with faecal slurry sepsis. Those animals had lower systemic cytokine and pro-coagulant responses, with upregulated endogenous endothelial nitric oxide synthase (eNOS) involved in circulatory function (Tyml et al., 2017). Short-term dietary restriction attenuated inflammatory response in adipose tissue and improved survival of middle-aged (12-month) septic mice (Starr et al., 2016).

Whilst caution must the taken when extrapolating LPS-induced effects observed in mice to human sepsis, due to, among others, the differing susceptibility to endotoxins (Fink, 2014) and fundamentally different characteristics and timing of the involved immune responses, they could be still useful models for assessing the potentially beneficial role of geroprotectors in boosting physical resilience.

## 3 Trauma

Although trauma has long been considered a leading cause of morbidity and mortality in the young and adult populations (Kong et al., 1996; Pfeifer et al., 2016), the Geriatric Trauma Committee (constituted by the American Association for the Surgery of Trauma) recently declared geriatric trauma as rapidly growing problem (Kozar et al., 2015). Older patients typically undergo blunt rather than penetrating trauma and the most common causes of geriatric trauma are falls and motor vehicle accidents (Bonne and Schuerer, 2013; Labib et al., 2011). Trauma patients with frailty are more likely to have in-hospital complications and have a higher incidence of mortality (Joseph et al., 2014a).

In contrast to any low-level persistent inflammatory conditions (e.g. arthritis), trauma constitutes a massive and sudden challenge to the host. The tissues and immuno-inflammatory system rapidly release a plethora of local and systemic mediators known as danger-associated molecular patterns (DAMPs) (Timmermans et al., 2016), which typically leads to systemic inflammatory response syndrome (SIRS) and subsequent sequelae. The overall resilience of the patient and their compensatory capability largely depends on the flexibility and adaptation mechanisms that control and regulate those responses. The magnitude of the impairment of those innate, adaptive, and homeostatic mechanisms defines the physiological reserve of the host and correlates with the subsequent complications and outcome (Banks and Lewis, 2013). For these reasons animal models of trauma could be valuable in testing the effects of geroprotectors in boosting physical resilience.

Due to high costs of maintenance and complexity, trauma studies in aged mice are infrequent. To date, responses of aged mice (i.e., 18 months of age and older) to trauma were investigated in models of burn (the most frequent) (Auger et al., 2017; Du et al., 2013; Koshizuka et al., 1997; Kovacs et al., 2002; Shallo et al., 2003; Swift et al., 2001), trauma (e.g. laparotomy, femur fracture) combined with hemorrhage (Drechsler et al., 2012; Kahlke et al., 2000a, 2000b; Schneider et al., 2006), traumatic brain injury (TBI) (Kumar et al., 2013; Morganti et al., 2016; Nakagawa et al., 2000) and more complex polytrauma settings (Kang et al., 2004; Matsutani et al., 2007; Nacionales et al., 2015). A recent review by Drechsler et al. (Drechsler et al., 2016) provides a detailed coverage of the available models of trauma in aged mice. Based on the current knowledge, trauma models such as burn and traumatic brain injury are preferable in testing resilience due to three main reasons: a) their set-up is relatively less complex compared with polytrauma models, b) severity adjustments are easier to perform (e.g., the amount of impact force used in TBI model, the total body surface area affected in burn model) and c) their post-injury sequelae are relatively protracted compared with other techniques such as laparotomy/hemorrhage.

In addition, these models have been shown to affect more than one tissue, with a more pronounced response in aged mice at least for some of the parameters measured. For example, there was an exaggerated activation and infiltration of monocytes/macrophages in the brain parenchyma in mice after TBI, which was greater in aged mice compared with young ones. The resulting enhanced local neuroinflammation and lesions (Kumar et al., 2013; Morganti et al., 2016) were associated with increased cognitive dysfunction (Morganti et al., 2015). In addition, atrophy and muscle wasting was described, although only in young mice following TBI (Shahidi et al., 2018). In the model of burn, young animals experienced a profound and prolonged loss of lean body mass, fat mass, and bone mineral density, associated with significant morbidity that was concurrent with the robust hypermetabolic response (Pedroso et al., 2012). In aged animals, pathological analysis of the lung showed an increased neutrophil infiltration compared with young animals (Nomellini et al., 2009), suggesting a stronger inflammatory response similar to that seen in older patients leading to lung failure and increased mortality. Indeed, after burn injury, aged mice exhibited higher mortality and produced more interleukin-6 when compared with young adult mice subjected to the same size injuries (Kovacs et al., 2002; Nomellini et al., 2009).

When modelling resilience in mice using trauma models, a number of points have to be considered (Li et al., 2017; Osuchowski et al., 2014). Rodents in general, and the mouse in particular, are more resistant to a variety of injuries compared with humans. This is at least partly due to a number of known species-inherent discrepancies within the immuno-inflammatory systems between mouse and man (Li et al., 2017; Mestas and Hughes, 2004; Osuchowski et al., 2014). For example, there are substantial differences in the composition of circulating leukocytes (Mestas and Hughes, 2004) and in the expression of some genes when comparing the same leukocytes subpopulations between humans and mice (Gentile et al., 2014; Mestas and Hughes, 2004; Shay et al., 2013). This implies that immune responses may differ and a careful evaluation of the investigated pathways needs to be considered in the trauma models employed. The development of more advanced (i.e. more severe) polytrauma models, which better recapitulate events observed in trauma patients may improve the translation of data from mouse to human (Mira et al., 2018), although such an approach presents serious welfare concerns. Furthermore, even in the event that the mouse models adequately match the pathophysiological responses of patients exposed to trauma, they are unlikely to be exposed to the intensive care unit (ICU) type clinical care (e.g., mechanical ventilation, blood transfusions, and invasive central lines) (Hartmann et al., 2018). These interventions are likely to interfere with the response to trauma and their absence precludes the assessment of endpoints such as quality of life, ventilator-free days and/or length of the ICU stay. Finally, clinical-like scoring systems focused on resilience/frailty need to be developed and used for rodent (and other) studies. Although not yet widely used, examples of such scores in mouse critical care models exist (Matute-Bello et al., 2011; Rademann et al., 2017; Shrum et al., 2014). A simplified trauma-specific frailty index (Joseph et al., 2014b), for example based on 10–15 relevant variables adapted for preclinical use, would allow better standardization and comparison of results across labs and studies.

## 4 Exposure to chemicals and radiation

There are a number of stressors which will result in loss of resilience in ageing mice such as administration of anaesthesia (reviewed in Kirkland et al., 2016) or exposure to chemicals causing oxidative damage such as Paraquat (reviewed in Kirkland et al., 2016). Here we will focus on effects of polypharmacy, chemotherapy, and radiotherapy.

### 4.1 Polypharmacy

Polypharmacy is a well-known problem in older people and it is a well-documented cause of increased hospitalization and mortality (Jyrkkä et al., 2009). Individuals with frailty are more sensitive to drugs and there is increased report of drug adverse events (Salvi et al., 2012). Huizer-Pajkos and colleagues reported findings from a novel polypharmacy mouse model (Huizer-Pajkos et al., 2016), in which mice were administered therapeutic doses of five commonly used medicines by older people (simvastatin, metoprolol, omeprazole, acetaminophen, and citalopram) for two to four weeks. When administered individually, the medicines had been shown to have no effect on performance in young mice. However, administered together they impaired physical function (open field test, rotarod, grip strength, and gait speed) in old but not in young mice. A more in-depth characterization of the loss of function experienced by the mice across systems in addition to the use of different combination of drugs to mimic polypharmacy is required. It will be interesting to test whether pre-treatment with geroprotectors is able to prevent the negative effects. If this is the case, this model is relatively easy to implement, not excessively taxing for the animals welfare, and is clinically relevant.

### 4.2 Chemotherapy and radiotherapy

More than half of older patients with cancer have frailty or prefrailty (Ethun et al., 2017). Frailty in older cancer patients affects outcomes due to the increased risk of treatment with surgery, chemotherapy, and radiotherapy. The cancer itself, as well as the therapies offered, are significant additional stressors that challenge the ability of patients to recover. Their reduced resilience means that they often never completely recover from the stress of surgery and chemotherapy with late effects and reduced quality of life (Collins et al., 2017). For these reasons, these groups of patients offer unique opportunities to test the effects of geroprotectors to boost physical resilience to cancer and its treatment, reduce late effects, and improve outcomes.

Extensive evidence of efficacy in animal studies are required before this intervention could be trialled in humans. Therefore, pre-clinical frail mouse models of cancer challenged by surgery and/or chemotherapy or radiotherapy are needed in order to test recovery from these stressors following treatment with geroprotectors. There are, however, very few examples of such models. Chemotherapy induced fatigue was assessed in 10-16-week-old p16-3MR female mice (Demaria et al., 2017). These were developed to contain functional domains of Renilla luciferase (LUC), and a truncated herpes simplex virus (HSV)-1 thymidine kinase (tTK) under control of the senescence-sensitive *p16^INK4a^* promoter, allowing tracking of senescence cells and their elimination via the administration of ganciclovir (GCV). Mice were treated with doxorubicin (Doxo) at a single dose of 10 mg/kg. This dose appeared to be biologically effective in that it was able to induce toxicity (e.g., myelosuppression) as well as an anti-tumor response but was below the maximally tolerated dose. Acute toxicity (excessive weight loss, rough fur, and inactivity) was evident at the highest dose of 25 mg/kg. While doses over 60 mg/kg are lethal in adult mice (Matsumura et al., 1994), the dose of 10 mg/kg is equivalent to the cumulative dose given to human patients who receive six to eight biologically effective doses of Doxo at 1.25 mg/kg with cumulative toxicity observed at 12.5 mg/kg. This procedure is a good compromise between developing models, which accurately reproduce the human condition, and remaining mindful of animal welfare.

Using running wheels in standard cages, Doxo-treated animals experienced a ~50% decline in running activity and a reduction in grip strength and weight loss, all signs associated with frailty. No changes in food consumption were observed to explain the weight loss. Elimination of senescent cells by GCV or ABT-263 after chemotherapy limited the reduction in running capacity to ~20% and improved strength as measured by their ability to grasp to the cage lid. However, there was no effect on the weight loss.

Whilst these experiments are encouraging in demonstrating that the effects of geroprotectors can be tested in such models and may be useful in boosting resilience, there is a need to conduct these experiments in age-appropriate animals. The dose of drugs employed in these studies may not be suitable because of the altered pharmacokinetics in aged animals. For example, in aged rats ketamine availability was 6–7 times greater than in young rats (Veilleux-Lemieux et al., 2013). In addition, tissues may not have as much regenerative capacity in aged animals. Finally, cancer develops slower but shows higher levels of metastatization in aged animals (Han et al., 2016). This impacts on the general wellbeing of the animal and the capacity to respond.

Chemotherapy has been administered to aged animals. An example is the administering of 5-Fluoruracil (5-FU) to the telomerase knock out mouse TR^−/−^ (a model of accelerated ageing) at 16–22 months old (Rudolph et al., 1999). A decline in all peripheral blood cells was observed - with the lowest point occurring six days after injection - as well as loss of body weight. This was more pronounced in aged G6 compared to aged mTR^+/+^, while G3 animals exhibited an intermediate phenotype. The clinical appearance and general condition of the aged G3 and G6 animals was markedly compromised. These results demonstrate a diminished capacity of the aged telomerase-deficient mice to respond to a stress known to challenge the regenerative potential of the hematopoietic and gastrointestinal systems. 5-FU administration in young and aged wild type mice led to selective cognitive deficits affecting executive functions, in particular cognitive flexibility and inhibitory control. However, these deficits were not worsened in aged animals, which displayed reduced performance of spatial learning, behavioural flexibility and object recognition memory already in absence of chemotherapy (Dubois et al., 2014). Cisplatin was also administered to aged animals and young animals were more resilient to cisplatin-induced nephrotoxicity (Wen et al., 2015). However, no assessment of several other systems such as mobility, endurance, and coordination were performed in any of these models to compare with the clinical situation. In addition, no treatment of any geroprotector was used to see whether any improvement was detectable.

Studies with radiation go back as far as the 1950s and show that sensitivity to radiation is more pronounced during growth and development before decreasing in adulthood then increasing once more in the later stages of life (Storer, 1962). For example, in 5-week-old 101xC3H F1 mice LD50/80 is 580 rads, while at 15 weeks it is 700 rads. However, sensitivity decreased again in mice aged over 400 days and the timing and dose sensitivity depended on the strain. Kohn and Kallman found that LD50 was 550 rads for BALB/c and 665 rads for C3H (Kohn and Kallman, 1956). Whilst these studies are informative, their aim was to gain an understanding of the effects of high-dose radiation on longevity. Therefore, they have limited utility as models of resilience.

Nevertheless, these data show that it is possible to develop models of physical resilience suitable for testing geroprotectors by challenging ageing animals with chemicals and radiation. Several drugs in clinical use, which are either already known to induce premature ageing or show severe side effects in ageing compared to young patients, such as platinum-based antineoplastic drugs, cyclophosphamide, Mitomycin C, specific inhibitors of BRAF/MEK, cell cycle regulators, as well as highly active antiretroviral therapy (HAART) may offer alternative opportunities. Although clinically relevant and thereby highly promising for physical resilience testing, they should first be assessed comprehensively across the five main systems (musculoskeletal, immunological, cognitive, metabolic, and cardiovascular) in ageing mice in order to determine their suitability. The question remains whether testing should occur in aged mice harbouring cancer. This may be difficult to achieve. Most models of cancer are very aggressive and develop very rapidly, leading to a fast decline of the mouse health. Therefore, the efficacy of the chemotherapy on the cancer and its negative effects on the physical resilience of mice may be best tested separately.

## 5 Ischemia

Ageing of vascular endothelial and smooth muscle cells induces progressive structural and functional changes of the vascular phenotype that may deteriorate the health of the whole organism. Arterial stiffness, oxidative stress, and inflammation are hallmarks of vascular ageing leading to hypertension, increased vascular permeability, atherosclerosis, and other vascular disturbances (Laina et al., 2018; Mistriotis and Andreadis, 2017). In addition to heart, both kidneys and brain are organs with high blood flow regulation and which are sensitive to vascular damage that can occur from increased aortic stiffness with advanced age (Cooper and Mitchell, 2016; Mitchell, 2008) potentially resulting in ischemic injury.

### 5.1 Renal ischemic injury

As with every system or organ in the body, ageing has a profound effect on renal function, which in turn can have a detrimental effect on other organs and systems. Chronic kidney disease is associated with an increased incidence of frailty (Musso et al., 2015; Shen et al., 2017; Walker et al., 2014) and acute kidney injury (AKI) is independently associated with increased frailty after critical illness (Abdel-Kader et al., 2018). Chronic kidney disease is also now emerging as a risk factor for cognitive decline and dementia, as well as its known association with cardiovascular disease (Elias et al., 2013; Miwa et al., 2014). Frail patients with kidney disease are more likely to decline but show greater improvement after kidney transplant than non-frail patients (McAdams-DeMarco et al., 2018), thus the resilience of the kidney can influence the overall frailty of a patient.

Whilst renal ischemic injury can occur spontaneously in aged kidneys, it can be recapitulated through surgical induction of ischemia, thus providing a platform for the standardised study of the process involved and interventions to modulate it. Bilateral renal ischemic injury in mice is a commonly used model for acute kidney injury research although the mouse model is less stable than others (e.g., the rat model) and requires skilled surgical practise (Wei and Dong, 2012). Studies in rats have shown increased damage and failure to recover after surgically induced ischemia in aged animals (Kusaka et al., 2012; Miura et al., 1987; Xu et al., 2014). Ischemic damage resulted in increased levels of blood urea nitrogen in both young and old rats but impaired recovery as highly increased levels in old animals persisted, whereas they returned to normal in young rats. Histopathological changes also persisted in old rats, suggesting a reduced capacity to repair damage (Kusaka et al., 2012; Miura et al., 1987; Xu et al., 2014). In mice, such studies in aged animals have only just started to emerge but show that old mice have a higher proportion of damaged tubules compared with young mice at least in the initial stages of acute renal ischemia (Jang et al., 2016).

There is no data as to whether acute kidney ischemia negatively affects other function such as memory and cognition and more so in aged animals. Muscle wasting has been reported in mice in a model of renal failure (Wang and Mitch, 2013). This is obtained following removal of the kidney following kidney ischemia. Until more work is done to characterise this model appropriately it is not possible to say whether this is a suitable model of physical resilience.

### 5.2 Brain ischemia

In the brain, presence of subclinical ischemic-like white matter lesions and cerebral vascular damage was reported in frail older persons compared with non-frail aged-matched individuals (Avila-Funes et al., 2017). In addition, frailty was associated with the incidence of vascular dementia (Avila-Funes et al., 2012). The most common cerebrovascular disease in the aged population is stroke. Ischemic stroke is more frequent (87% of total cases) than hemorrhagic stroke (10% intracerebral hemorrhage and 3% subarachnoid hemorrhage) (Mozaffarian et al., 2015). Ischemic stroke is caused by deficit of local blood flow resulting in brain lesions and cell death in the affected areas, either grey matter or white matter. Pathologic mechanisms of ischemia and subsequent reperfusion injury involve excitotoxicity, increased generation of free radicals, and an inflammatory response. Furthermore, increased BBB permeability with leukocyte infiltration and cerebral edema impairs recovery processes. The cascade of events eventually lead to necrotic or apoptotic cell death (Karsy et al., 2017). In the hemorrhagic stroke there is the release of cytotoxic hemoglobin that increases oxidative damage (Aronowski and Zhao, 2011).

Stroke may induce early systemic effects that increase the frailty status and thereby delay the recovery of the patient. Stroke-induced sarcopenia derives from innervation changes, peripheral inflammation, and metabolic processes that cause loss of skeletal muscle tissue (Scherbakov et al., 2013). There is an acute post-stroke hyperglycemia as an adaptive stress response to ischemia, although hyperglycemia is considered deleterious for the metabolic processes of ischemia-reperfusion (Robbins and Swanson, 2014) and is associated with poor clinical outcome (Chen et al., 2016). Systemic immunosuppression derived from inflammatory processes and apoptosis of immune cells is also frequent (Liu et al., 2017), increasing susceptibility to pneumonia and other infections (Shim and Wong, 2016).

Mouse models developed for studying cerebral ischemia might be adapted for testing cerebrovascular resilience in aged mice. We propose two highly characterized models. A widely used rodent model of focal brain ischemia and reperfusion is Middle Cerebral Artery Occlusion (MCAO), where left or right middle cerebral artery is temporarily occluded by a suture introduced through the internal carotid artery followed by reestablishment of blood flow (Liu et al., 2009). Occlusion time determines the severity of the outcome. The MCA is the vessel most commonly affected by cerebrovascular accident, which causes lesions in the cortex and striatum. Sensorimotor function is impaired in most animals and can be measured by standardized tests such as grip strength, rotarod, open field, pole test, cylinder asymmetry, and adhesive removal test. Furthermore, a neurological score can be obtained as a composite of motor, reflex, and balance scales (Balkaya et al., 2013; Freret et al., 2011). In young adult mice, a transient ischemia of 45–60 min caused mild lesions allowing functional motor recovery after 7–14 days, whereas those mice submitted to 90-min or permanent MCAO did not recover (Tachibana et al., 2017). As previously described for aged rats after MCAO (Popa-Wagner et al., 2011; Rosen et al., 2005), aged mice are more vulnerable to ischemic injury than younger ones and did not show functional improvement after a 60-min MCAO (Manwani et al., 2013; Zhou et al., 2017). Studies in aged mice are scarce, but we can assume that a shorter period of MCAO, such as less than 30 min, could be suitable for testing cerebrovascular resilience by measuring the progression of functional improvement seen in sensorimotor function after the ischemic stressor. In addition, other measures that might be relevant to frailty outcomes should be routinely introduced. This is the case for bodyweight control. Mice suffer significant loss of body weight after ischemia that parallels their functional impairment, with spontaneous recovery after 14–21 days in young adult mice (Park et al., 2014). In this mouse model, there is a loss of skeletal muscle tissue and therefore it reproduces the human post-stroke sarcopenia (Springer et al., 2014). More studies are needed to test for the presence of other stroke-induced systemic effects and to standardize the MCAO model of resilience in aged mice.

A mouse model of vascular cognitive impairment by chronic cerebral hypoperfusion is Bilateral Common Carotid Artery Stenosis (BCAS). Stenosis is induced by the surgical placement of microcoils around the arteries. The level of blood flow reduction is given by the size of the coil. BCAS mimics many aspects of the brain pathology underlying vascular dementia in humans (Bink et al., 2013; Holland et al., 2015). BCAS hypoperfusion cause diffuse ischemic lesions mainly in white matter, throughout the brain. Working memory deficits can be detected with the 8-arm radial maze test and Y maze test, at one to several months after BCAS establishment (Coltman et al., 2011; Maki et al., 2011). Deficits in recognition memory have also been reported (Patel et al., 2017). The most used coils of 0.18 mm decrease blood flow to 70% at two hours and it is partially recovered to 80% after one to three months. Coils of 0.20 mm induce milder peak effects to 80% blood flow that revert after one month, but the reproducibility is lower (Shibata et al., 2004). Cognitive deficits induced by BCAS by means of 0.18 mm coils are significantly more severe in aged mice than in younger ones, showing more severe lesions of myelin integrity and neuroinflammation (Wolf et al., 2017). Similarly to a MCAO-based resilience model, milder hypoperfusion lesion by means of using a coil with a higher diameter in aged mice could establish a more appropriate BCAS resilience model. However, BCAS is a recent model with scarcity of information in aged mice and preliminary studies would be needed to confirm the physical resilience testing reliability.

## 6 Noise

Noise is the most common cause of acquired deafness in developed countries and therefore it is a public health priority (Sliwinska-Kowalska and Davis, 2012). Irreversible noise-induced hearing loss (NIHL) can result from a single high-intensity exposure or by repetitive exposure to moderate or high-intensity noise. There is an individual susceptibility to noise with a genetic component that is not entirely well understood. Interestingly, there are gene polymorphisms associated with resistance to NIHL (Pawelczyk et al., 2009) as well as to NIHL predisposition (Konings et al., 2009; Le Prell, 2012). Importantly, noise exposure is also significantly associated with the impairment of several other non-auditory body functions, including elevated blood pressure in young middle-aged and old individuals (Chang et al., 2009), impaired immune activity, sleep disturbances (Prasher, 2009), increased risk of ischemic heart disease and cerebrovascular disease (Recio et al., 2016) and ultimately increased cardiovascular and respiratory mortality among people aged ≥65 years (Tobías et al., 2015).

The study of the pathophysiology of NIHL has been carried out mostly in mouse models. Mice have a hearing range in higher frequencies than humans, but the functional and structural alterations observed are similar. Test showed 21-month-old CBA/CaJ mice with near-normal initial hearing were more vulnerable to NIHL than younger mice (Miller et al., 1998; Ohlemiller, 2006). The level of noise, the duration of the exposure, the chronicity of exposure, among other physical factors, determine the level of injury (Park et al., 2013). It is interesting to note that there is also strain susceptibility; C57BL/6 J mice are more susceptible to damage, whereas the CBA/CaJ and CBA/J mouse strains are more resilient (Sanz et al., 2015). The basis underlying the aforementioned differences is still to be determined.

To expose mice to noise stress, conscious animals are placed in a sound reverberant chamber, and exposed to a violet swept sine noise (frequency range 2–20 kHz) at 110 dB for a short period of time (Sanz et al., 2015). The noise stimuli can be adjusted depending on the mouse strain. The auditory response is estimated by measuring the auditory brainstem response.

Little data is available with regard to the evaluation of the effects of noise exposure on non-auditory systems in mice. One study showed that peritoneal and alveolar macrophage function were altered by noise stress in 22–24 months old C57BL/6 mice (de Wazières et al., 1998). Another group reported an association between increased oxidative stress levels, decreased memory function and noise in young mice (Sikandaner et al., 2017). Exposure of C57BL/6 mice to repetitive patterns of aircraft noise at low volume for four days did not affect the auditory system, but increased blood pressure, stress hormone levels and oxidative stress levels, while vascular function was reduced (Münzel et al., 2017). Whilst more testing is required, exposure to noise may offer opportunities to develop models of physical resilience which are relatively simple to establish.

## 7 Circadian rhythms alterations

Throughout our bodies, a vast range of biological processes are modulated by an internal circadian clock. In the absence of external cues, this clock will maintain rhythmicity with a period of around 24 h. However, in order to function usefully the phase of this clock can be reset by a range of environmental signals in a process known as entrainment. In mammals, the most important external cue is the light-dark cycle of day and night, which is detected by retinal photo-receptors. There is strong evidence in humans that aberrant light cycles, generally over extended periods of months or more, can act as a stressor through the disruption of the circadian clock and that such disruption can result in adverse health effects (Foster and Wulff, 2005). Exposure to light at night is associated with elevated risks of cancer (Kloog et al., 2011), obesity (Wyse et al., 2011), and depression (Fonken and Nelson, 2013). Additionally, chronic shift work (in which circadian rhythms are out of phase with the day/night light cycle) has a number of negative impacts including reduced cognitive performance and elevated risks of cardiovascular disease and cancer (Evans and Davidson, 2013). Furthermore, it has been demonstrated that this kind of circadian disruption not only prematurely ages the brain, but also that it can take years to recover from prolonged bouts of shifts to the circadian cycle (Marquié et al., 2015).

While there is clear evidence for the deleterious effect of aberrant light-dark cycles on both health and healthy ageing, there are difficulties in modelling and assessing the stressor effects of such disruptions for several reasons. Young, 2-4-month old, mice subjected to acute jet-lag paradigms (in which animals undergo a single six-hour shift in the light cycle) show stress responses such as an elevated stress evoked corticosterone response and disrupted learning and memory (Loh et al., 2010). However, if mice are subjected to chronic jet-lag paradigms (in which animals undergo repeated six-hour shifts in the light cycle over a four-week period) baseline corticosterone and anxiety related behaviours are reported to be unaffected (Castanon-Cervantes et al., 2010). Furthermore, a study which subjected young and aged mice to chronic jet-lag for eight weeks, demonstrated that while chronic stress (as measured by faecal corticosterone levels) appeared to be unaffected across the two groups, the aged cohort showed increased mortality rates compared to the young cohort (Davidson et al., 2006). It therefore appears that the health consequences of altered light cycles are present even in the absence of commonly used hormonal or behavioural measures of stress, but it is unclear at present what parameters of health are altered.

An additional complication lies in the fact that the circadian disruption, resulting from altered light cycles, will not only affect the central circadian clock in the brain. Other tissues and organs also contain their own clocks, known as peripheral clocks. These clocks reentrain to light-dark cycle changes at different rates to the core clock, potentially leading to a situation where the peripheral and core clocks are out of phase while they re-synchronise (Yamazaki et al., 2000). Since loss of peripheral clock rhythmicity through deletion of clock genes has been shown to adversely affect health (Lamia et al., 2008), it is possible that phase discrepancies between the core and peripheral clocks may underpin the effects of changes to the light-dark cycle.

Thus, it is clear that disrupted light cycles can have adverse effects on health, and these are more pronounced in aged mice. However, in absence of a better understanding of the mechanisms undergoing dysregulation and a thorough characterization of each organ function following disruption, it is difficult to assess whether this challenge is suitable as model of physical resilience for geroprotector testing.

## 8 Temperature stress

### 8.1 Heat

Old age severely impairs the ability of humans to cope with changes of ambient temperature (Gagnon et al., 2016). This is clearly reflected by increased numbers of hospital admissions and deaths of older people during recent heat waves in France and Chicago (Fouillet et al., 2006; Whitman et al., 1997). Young and healthy individuals react to elevated ambient temperature by increased sweat production, skin temperature, and cardiac output, while this response is blunted even in healthy aged humans (Kenney and Munce, 2003).

Mice undergo similar systemic changes and young animals are better able to avoid hyperthermia during heat stress than old animals (Hoffman-Goetz and Keir, 1984). Repeated exposure to heat significantly decreases food consumption and body weight during the experimental period, while water intake is not affected (Harikai et al., 2003). The magnitude and length of recovery depends on the severity of the heat. Pathological changes in the lung and brain, such as chronic inflammation, ischemic, hypoxic, and oxidative damage have been observed upon severe heat stress (Tseng et al., 2014). These were associated with loss of memory and cognitive function (Lee et al., 2015), as well as with alterations in hormone levels, deficits in thermoregulation, and decreased survival (Tseng et al., 2014).

The technique is relatively simple and does not require anaesthesia, which may act as confounding factor. Before starting experimentation, it is important to adapt mice to their thermoneutral temperature of 30 °C for two to four weeks (Nguyen et al., 2011), as in most animal facilities rodents are housed at temperatures of 21–23 °C. Thereby, animals are constantly exposed to mild cold stress, which might affect accurate modelling of human homeostasis and disease (Karp, 2012), as well as several physiological parameters including the heart rate (Swoap et al., 2008). A recent study provides clear evidence that the energy expenditure of mice housed at 30 °C mimics the human situation much closer than mice housed at 21 °C (Fischer et al., 2018).

After this adaptation period, mice can be housed at elevated temperature or (partially) submerged in warm water to induce the actual heat stress. For exposure to dry heat, adapted steel cages keeping the temperature constant and homogeneously distributed during the incubation period are recommended. Physical restraining of animals is not required. Short incubation periods of 40 min at 42 °C (Vallanat et al., 2010) or three hours at 39.5 °C (Chen and Islam, 2016) seem most desirable for practical and animal welfare reasons, although also longer treatment regimens with exposure periods of 8–24 hours daily and recovery periods of between one day and six weeks were described (Houston et al., 2018). Body core temperature should be constantly monitored with implanted transponders or other temperature probes during heat exposure to terminate the experiment in case the body core temperature reaches 42.2 °C (Chen and Yu, 2017).

Several studies suggest that cellular heat shock responses decline during cellular and organismal ageing (reviewed by Calderwood et al., 2009) and peritoneal macrophages isolated from old mice recovered significantly slower than cells isolated from middle-aged or young animals after exposure to heat stress *in-vitro* (Lavie et al., 1992). However, direct experimental evidence for use of this stress in aged animals is still lacking and it is unknown whether aged animals are more sensitive to heat stress also on organismal level. Optimization of protocols in aged mice and a full characterization of the organ dysfunction needs to be performed. However, the simplicity of the technique and the changes observed in young animals seems to suggest promise for their use in testing the effects of geroprotectors on physical resilience.

### 8.2 Cold

Age also impairs the resilience to cold ambient temperatures. The response to cold exposure involves peripheral vasoconstriction to minimize heat loss, as well as heat production by non-shivering and shivering thermogenesis (Greaney et al., 2015). All three mechanisms become less functional with age: Vasoconstriction is diminished in old skin, while brown adipose tissue deposits required for non-shivering thermogenesis (Schosserer et al., 2018) and muscle function necessary for shivering thermogenesis, decline. These processes, together with less food intake and physical activity which are required for heat generation, cause an increase of accidental hypothermia and delayed recovery from cold stress in aged humans (Horvath and Rochelle, 1977; Vaughan et al., 1981).

Aged mice and rats are also less able to maintain their body core temperature during cold shock exposure and recover slower from this stressor (Balmagiya and Rozovski, 1983; Schorr et al., 2018; Shefer et al., 1996). In mouse experiments conducted by Schorr et al, animals were submerged in a water bath at 22 °C for 10 min, wiped dry and allowed to recover at 30 °C. Body temperature was monitored constantly by an implanted digital transponder in order to avoid strong hypothermia (Tc < 32 °C) during the treatment period (Schorr et al., 2018).

In most other reports cold shock response was assessed only in young mice by exposing animals to 4–10 °C ambient temperature for three to ten hours, while monitoring colonic temperature in short intervals throughout the period of cold exposure (Talan and Engel, 1984; Talan et al., 1985; Tatelman and Talan, 1990; Vatner et al., 2018). In some cases, mice were physically restrained during cold exposure to minimize movement, which might confound experimental outcomes (Talan and Engel, 1984; Talan et al., 1985; Tatelman and Talan, 1990).

In humans and rodents, hypothermia frequently occurs during anaesthesia and has serious consequences that include depressed cardiac and respiratory function, delayed anaesthetic recovery, decreased wound healing, altered pharmacokinetics and diminished efficacy of the immune response (Sessler, 2001; Skorupski et al., 2017). Although experimental evidence that these physiological functions are also affected in non-sedated and aged animals by cold exposure is still lacking, they still might be considered as suitable readouts when developing a cold-shock model of physical resilience.

## 9 Future directions

Although it is well known that the decline of physical resilience with age is one of the major causes of frailty and ageing-associated pathology in humans, the definition and criteria to select a model of physical resilience is challenging. There is much need for discussion and consensus around whether it is sufficient to focus on stresses that produce a delayed recovery in older mice only in one specific tissue or whether it is necessary to apply more stringent criteria and look for stresses, which cause delayed recovery of multiple deficits. We have decided to adopt models, which, at least in principle, conform to the second definition as it was felt this is more clinically relevant and reflects the nature of frailty in terms of accumulation of deficits. However, most of these stresses are very serious in terms of animal welfare. In addition, there is very little data related to the characterization of the multiple deficits caused, especially in aged animals. The list of models in this review is not exhaustive. For example, with more research, models of would healing or Bone fracture may offer opportunities but at present there is too little data to draw any conclusion. More data are required together with robust discussions between researchers on ageing and geriatricians to reach consensus on the suitability of such models to support efficacy of geroprotectors to boost physical resilience in frail individuals.

## Figures and Tables

**Table 1:** Summary of stressors discussed in this review with rating for key evidence with regard to the effect of age on the response to the stressor (* very poor, *** good, none of them is excellent), to the effects on multiple systems which would suggest the potential to test effects on integrated responses (* very poor, *** good, none being exellent), severity of the procedure in terms of animal welfare and clinical relevance a stressor associated with loss of resilience in patients with frailty. No model stands out as preferable. More characterization is needed before drawing any conclusion. The use of multiple models may be required as each one of them cover spcific aspects.

Stressor	Method	Strength of evidence for slower recovery with age	Strength of evidence for effects on multiple systems	Severity for animal welfare	Clinical relevance for resilience	Specialized equipment required	Surgical training required	References
Sepsis	Endotoxin/LPS injection	***	***	Moderate/severe	*	no	no	Saito et al. (2003); Tateda et al. (1996)
	i.p. injection of fecal slurry	none	**	Moderate/severe	**	no	no	Tyml et al. (2017)
	Cecal ligation and puncture	none	**	Moderate/severe	**	no	yes	Dejager et al. (2011)
trauma	Burn	***	***	Moderate/Severe	**	no	no	Auger et al. (2017); Shallo et al. (2003); Du et al. (2013)
	Laparotomy/hemorrhage	**	**	Moderate/Severe	**	no	yes	Drechsler et al. (2012); Kahlke et al.(2000a,b); Schneider et al. (2006)
	Traumatic brain injury	***	***	Severe	**	yes	yes	Kumar et al. (2013); Morganti et al. (2016); Nakagawa et al. (2000)
Exposure to chemicals and radiation	polypharmacy	***	**	Mild/moderate	***	no	no	Huizer-Pajkos et al. (2016)
	Doxorubicin	None	**	Mild/moderate	**	no	no	Demaria et al. (2017)
	5-Fluoruracil	**	**	Mild/moderate	**	no	no	Rudolph et al. (1999)
	radiation (e.g. X-rays)	**	**	Mild/moderate	**	yes	no	Kohn and Kallman (1956); Storer (1962)
kidney ischemia	Bilateral renal ischemia injury	**	*	Moderate/severe	*	no	yes	Wei and Dong (2012); Jang et al. (2016)
brain ischemia	Middle Cerebral Artery Occlusion	**	**	Moderate	***	no	yes	Liu et al. (2009); Manwani et al. (2013); Zhou et al. (2017)
	Bilateral Common Carotid Artery Stenosis	**	None	Moderate	***	no	yes	Bink et al. (2013); Holland et al. (2015); Wolf et al. (2017)
noise	Exposure to violet swept sine noise or simulated airplane noise	**	*	Mild	*	yes	no	Miller et al., (1998); Park et al. (2013); Sanz et al. (2015)
light-dark cycles	light cycle shift (jet-lag)	**	*	Mild	*	no	no	Loh et al. (2010); Davidson et al. (2006); Castanon-Cervantes et al. (2010)
heat stress	increase of ambient temperature	**	*	Mild	**	yes	no	Vallanat et al. (2010); Chen and Islam (2016); Chen and Yu (2017)
cold stress	immersion in cold water bath & decrease of ambient temperature	*	*	Mild/moderate	**	no	no	Schorr et al. (2018) Shefer et al. (1996); Tatelman and Talan (1990); Vatner et al. (2018)

## References

[R1] Abdel-Kader K, Girard TD, Brummel NE, Saunders CT, Blume JD, Clark AJ, Vincz AJ, Ely EW, Jackson JC, Bell SP, Archer KR (2018). Acute kidney injury and subsequent frailty status in survivors of critical illness: a secondary analysis. Crit Care Med.

[R2] Aronowski J, Zhao X (2011). Molecular pathophysiology of cerebral hemorrhage: secondary brain injury. Stroke.

[R3] Auger C, Sivayoganathan T, Abdullahi A, Parousis A, Jeschke MG (2017). Hepatic mitochondrial bioenergetics in aged C57BL/6 mice exhibit delayed recovery from severe burn injury. Biochim Biophys Acta.

[R4] Avila-Funes JA, Carcaillon L, Helmer C, Carrière I, Ritchie K, Rouaud O, Tzourio C, Dartigues J-F, Amieva H (2012). Is frailty a prodromal stage of vascular dementia? Results from the Three-City study. J Am Geriatr Soc.

[R5] Avila-Funes JA, Pelletier A, Meillon C, Catheline G, Periot O, Trevin O-Frenk I, Gonzalez-Colaço M, Dartigues J-F, Pérès K, Allard M, Dilharreguy B (2017). Vascular cerebral damage in frail older adults: the AMImage study. J Gerontol A Biol Sci Med Sci.

[R6] Balkaya M, Kröber JM, Rex A, Endres M (2013). Assessing post-stroke behavior in mouse models of focal ischemia. J Cereb Blood Flow Metab.

[R7] Balmagiya T, Rozovski SJ (1983). Age-related changes in thermoregulation in male albino rats. Exp Gerontol.

[R8] Banks SE, Lewis MC (2013). Trauma in the elderly: considerations for anesthetic management. Anesthesiol Clin.

[R9] Bellantuono I (2018). Find drugs that delay many diseases of old age. Nature.

[R10] Bink DI, Ritz K, Aronica E, van der Weerd L, Daemen MJAP (2013). Mouse models to study the effect of cardiovascular risk factors on brain structure and cognition. J Cereb Blood Flow Metab.

[R11] Bonne S, Schuerer DJE (2013). Trauma in the older adult: epidemiology and evolving geriatric trauma principles. Clin Geriatr Med.

[R12] Calabrese EJ, Bachmann KA, Bailer AJ, Bolger PM, Borak J, Cai L, Cedergreen N, Cherian MG, Chiueh CC, Clarkson TW, Cook RR (2007). Biological stress response terminology: integrating the concepts of adaptive response and preconditioning stress within a hormetic dose-response framework. Toxicol Appl Pharmacol.

[R13] Calderwood SK, Murshid A, Prince T (2009). The shock of aging: molecular chaperones and the heat shock response in longevity and aging–a mini-review. Gerontology.

[R14] Castanon-Cervantes O, Wu M, Ehlen JC, Paul K, Gamble KL, Johnson RL, Besing RC, Menaker M, Gewirtz AT, Davidson AJ (2010). Dysregulation of inflammatory responses by chronic circadian disruption. J Immunol.

[R15] Chang T-Y, Lai Y-A, Hsieh H-H, Lai J-S, Liu C-S (2009). Effects of environmental noise exposure on ambulatory blood pressure in young adults. Environ Res.

[R16] Chen Y, Islam A (2016). Transgenic sickle cell trait mice do not exhibit abnormal thermoregulatory and stress responses to heat shock exposure. Blood Cells Mol Dis.

[R17] Chen Y, Yu T (2017). Glucocorticoid receptor activation is associated with increased resistance to heat-induced hyperthermia and injury. Acta Physiol (Oxf).

[R18] Chen R, Ovbiagele B, Feng W (2016). Diabetes and stroke: epidemiology, pathophysiology, pharmaceuticals and outcomes. Am J Med Sci.

[R19] Chung KW, Lee EK, Kim DH, An HJ, Kim ND, Im DS, Lee J, Yu BP, Chung HY (2015). Age-related sensitivity to endotoxin-induced liver inflammation: implication of inflammasome/IL-1β for steatohepatitis. Aging Cell.

[R20] Collins K, Reed M, Lifford K, Burton M, Edwards A, Ring A, Brain K, Harder H, Robinson T, Cheung KL, Morgan J (2017). Bridging the age gap in breast cancer: evaluation of decision support interventions for older women with operable breast cancer: protocol for a cluster randomised controlled trial. BMJ Open.

[R21] Coltman R, Spain A, Tsenkina Y, Fowler JH, Smith J, Scullion G, Allerhand M, Scott F, Kalaria RN, Ihara M, Daumas S (2011). Selective white matter pathology induces a specific impairment in spatial working memory. Neurobiol Aging.

[R22] Cooper LL, Mitchell GF (2016). Aortic stiffness, cerebrovascular dysfunction, and memory. Pulse (Basel, Switzerland).

[R23] Coopersmith CM, Stromberg PE, Dunne WM, Davis CG, Amiot DM, Buchman TG, Karl IE, Hotchkiss RS (2002). Inhibition of intestinal epithelial apoptosis and survival in a murine model of pneumonia-induced sepsis. JAMA.

[R24] Cunningham C, Hennessy E (2015). Co-morbidity and systemic inflammation as drivers of cognitive decline: new experimental models adopting a broader paradigm in dementia research. Alzheimers Res Ther.

[R25] Davidson AJ, Sellix MT, Daniel J, Yamazaki S, Menaker M, Block GD (2006). Chronic jet-lag increases mortality in aged mice. Curr Biol.

[R26] de Wazières B, Spehner V, Harraga S, Laplante F, Corallo F, Bloy C, Dupond JL, Vuitton DA, Seillès E (1998). Alteration in the production of free oxygen radicals and proinflammatory cytokines by peritoneal and alveolar macrophages in old mice and immunomodulatory effect of RU 41740 administration. Part I: effect of short and repetitive noise stress. Immunopharmacology.

[R27] Dejager L, Pinheiro I, Dejonckheere E, Libert C (2011). Cecal ligation and puncture: the gold standard model for polymicrobial sepsis?. Trends Microbiol.

[R28] Demaria M, O’Leary MN, Chang J, Shao L, Liu S, Alimirah F, Koenig K, Le C, Mitin N, Deal AM, Alston S (2017). Cellular senescence promotes adverse effects of chemotherapy and cancer relapse. Cancer Discov.

[R29] Drechsler S, Weixelbaumer K, Raeven P, Jafarmadar M, Khadem A, van Griensven M, Bahrami S, Osuchowski MF (2012). Relationship between age/gender-induced survival changes and the magnitude of inflammatory activation and organ dysfunction in post-traumatic sepsis. PLoS One.

[R30] Drechsler S, Lynch MA, Novella S, González-Navarro H, Hecimovic S, Barini E, Tucci V, Castro RE, Vandenbroucke RE, Osuchowski M, Potter PK (2016). With mouse age comes wisdom: a review and suggestions of relevant mouse models for age-related conditions. Mech Ageing Dev.

[R31] Du J, Liu L, Lay F, Wang Q, Dou C, Zhang X, Hosseini SM, Simon A, Rees DJ, Ahmed AK, Sebastian R (2013). Combination of HIF-1α gene transfection and HIF-1-activated bone marrow-derived angiogenic cell infusion improves burn wound healing in aged mice. Gene Ther.

[R32] Dubois M, Lapinte N, Villier V, Lecointre C, Roy V, Tonon M-C, Gandolfo P, Joly F, Hilber P, Castel H (2014). Chemotherapy-induced long-term alteration of executive functions and hippocampal cell proliferation: role of glucose as adjuvant. Neuropharmacology.

[R33] Elias MF, Dore GA, Davey A (2013). Kidney disease and cognitive function. Contrib Nephrol.

[R34] Ethun CG, Bilen MA, Jani AB, Maithel SK, Ogan K, Master VA (2017). Frailty and cancer: implications for oncology surgery, medical oncology, and radiation oncology. CA Cancer J Clin.

[R35] European Commission (2009). European Economy No. 2/2009 — the 2009 Ageing Report: Economic and Budgetary Projections for the EU-27 Member States (2008-2060).

[R36] Evans JA, Davidson AJ (2013). Health consequences of circadian disruption in humans and animal models. Prog Mol Biol Transl Sci.

[R37] Ferrucci L, Guralnik JM, Studenski S, Fried LP, Cutler GB, Walston JD, Interventions on Frailty Working Group (2004). Designing randomized, controlled trials aimed at preventing or delaying functional decline and disability in frail, older persons: a consensus report. J Am Geriatr Soc.

[R38] Figueira I, Fernandes A, Mladenovic Djordjevic A, Lopez-Contreras A, Henriques CM, Selman C, Ferreiro E, Gonos ES, Trejo JL, Misra J, Rasmussen LJ (2016). Interventions for age-related diseases: shifting the paradigm. Mech Ageing Dev.

[R39] Fink MP (2014). Animal models of sepsis. Virulence.

[R40] Fischer AW, Cannon B, Nedergaard J (2018). Optimal housing temperatures for mice to mimic the thermal environment of humans: an experimental study. Mol Metab.

[R41] Fonken LK, Nelson RJ (2013). Dim light at night increases depressive-like responses in male C3H/HeNHsd mice. Behav Brain Res.

[R42] Foster RG, Wulff K (2005). The rhythm of rest and excess. Nat Rev Neurosci.

[R43] Fouillet A, Rey G, Laurent F, Pavillon G, Bellec S, Guihenneuc-Jouyaux C, Clavel J, Jougla E, Hémon D (2006). Excess mortality related to the August 2003 heat wave in France. Int Arch Occup Environ Health.

[R44] Freret T, Schumann-Bard P, Boulouard M, Bouet V (2011). On the importance of long-term functional assessment after stroke to improve translation from bench to bedside. Exp Transl Stroke Med.

[R45] Gagnon D, Romero SA, Ngo H, Sarma S, Cornwell WK, Poh PYS, Stoller D, Levine BD, Crandall CG (2016). Healthy aging does not compromise the augmentation of cardiac function during heat stress. J Appl Physiol.

[R46] Gentile LF, Nacionales DC, Lopez MC, Vanzant E, Cuenca A, Cuenca AG, Ungaro R, Baslanti TO, McKinley BA, Bihorac A, Cuschieri J (2014). A better understanding of why murine models of trauma do not recapitulate the human syndrome. Crit Care Med.

[R47] Ginde AA, Moss M, Shapiro NI, Schwartz RS (2013). Impact of older age and nursing home residence on clinical outcomes of US emergency department visits for severe sepsis. J Crit Care.

[R48] Greaney JL, Alexander LM, Kenney WL (2015). Sympathetic control of reflex cutaneous vasoconstriction in human aging. J Appl Physiol.

[R49] Hadley EC, Kuchel GA, Newman AB, Workshop Speakers and Participants (2017). Report: NIA workshop on measures of physiologic resiliencies in human aging. J Gerontol A Biol Sci Med Sci.

[R50] Han Z, Brown JR, Niederkorn JY (2016). Growth and metastasis of intraocular tumors in aged mice. Investig Opthalmol Vis Sci.

[R51] Harikai N, Tomogane K, Miyamoto M, Shimada K, Onodera S, Tashiro S (2003). Dynamic responses to acute heat stress between 34 degrees C and 38.5 degrees C, and characteristics of heat stress response in mice. Biol Pharm Bull.

[R52] Hartmann C, Gröger M, Noirhomme J-P, Scheuerle A, Möller P, Wachter U, Huber-Lang M, Nussbaum B, Jung B, Merz T, McCook O (2018). In-depth characterization of the effects of cigarette smoke exposure on the acute trauma response and hemorrhage in mice. Shock.

[R53] Hoffman-Goetz L, Keir R (1984). Body temperature responses of aged mice to ambient temperature and humidity stress. J Gerontol.

[R54] Holland PR, Searcy JL, Salvadores N, Scullion G, Chen G, Lawson G, Scott F, Bastin ME, Ihara M, Kalaria R, Wood ER (2015). Gliovascular disruption and cognitive deficits in a mouse model with features of small vessel disease. J Cereb Blood Flow Metab.

[R55] Horvath SM, Rochelle RD (1977). Hypothermia in the aged. Environ Health Perspect.

[R56] Houston BJ, Nixon B, Martin JH, Iuliis GNDE, Bromfield EG, McEwen KE, Aitkena RJ (2018). Heat exposure induces oxidative stress and DNA damage in the male germ line. Biol Reprod.

[R57] Huizer-Pajkos A, Kane AE, Howlett SE, Mach J, Mitchell SJ, de Cabo R, Le Couteur DG, Hilmer SN (2016). Adverse geriatric outcomes secondary to polypharmacy in a mouse model: the influence of aging. J Gerontol A Biol Sci Med Sci.

[R58] Iwashyna TJ, Ely EW, Smith DM, Langa KM (2010). Long-term cognitive impairment and functional disability among survivors of severe sepsis. JAMA.

[R59] Jang HR, Park JH, Kwon GY, Park JB, Lee JE, Kim DJ, Kim Y-G, Kim SJ, Oh HY, Huh W (2016). Aging has small effects on initial ischemic acute kidney injury development despite changing intrarenal immunologic micromilieu in mice. Am J Physiol Renal Physiol.

[R60] Joseph B, Pandit V, Zangbar B, Kulvatunyou N, Hashmi A, Green DJ, O’Keeffe T, Tang A, Vercruysse G, Fain MJ, Friese RS (2014a). Superiority of frailty over age in predicting outcomes among geriatric trauma patients: a prospective analysis. JAMA Surg.

[R61] Joseph B, Pandit V, Zangbar B, Kulvatunyou N, Tang A, O’Keeffe T, Green DJ, Vercruysse G, Fain MJ, Friese RS, Rhee P (2014b). Validating trauma-specific frailty index for geriatric trauma patients: a prospective analysis. J Am Coll Surg.

[R62] Jyrkkä J, Enlund H, Korhonen MJ, Sulkava R, Hartikainen S (2009). Polypharmacy status as an indicator of mortality in an elderly population. Drugs Aging.

[R63] Kahlke V, Angele MK, Ayala A, Schwacha MG, Cioffi WG, Bland KI, Chaudry IH (2000a). Immune dysfunction following trauma-haemorrhage: influence of gender and age. Cytokine.

[R64] Kahlke V, Angele MK, Schwacha MG, Ayala A, Cioffi WG, Bland KI, Chaudry IH (2000b). Reversal of sexual dimorphism in splenic T lymphocyte responses after trauma-hemorrhage with aging. Am J Physiol Cell Physiol.

[R65] Kang S-C, Matsutani T, Choudhry MA, Schwacha MG, Rue LW, Bland KI, Chaudry IH (2004). Are the immune responses different in middle-aged and young mice following bone fracture, tissue trauma and hemorrhage?. Cytokine.

[R66] Karp CL (2012). Unstressing intemperate models: how cold stress undermines mouse modeling. J Exp Med.

[R67] Karsy M, Brock A, Guan J, Taussky P, Kalani MYS, Park MS (2017). Neuroprotective strategies and the underlying molecular basis of cerebrovascular stroke. Neurosurg Focus.

[R68] Kenney WL, Munce TA (2003). Invited review: aging and human temperature regulation. J Appl Physiol.

[R69] Kirkland JL, Stout MB, Sierra F (2016). Resilience in aging mice. J Gerontol A Biol Sci Med Sci.

[R70] Kloog I, Portnov BA, Rennert HS, Haim A (2011). Does the modern urbanized sleeping habitat pose a breast cancer risk?. Chronobiol Int.

[R71] Kohn HI, Kallman RF (1956). Age, Growth, and the LD50 of X-rays. Science.

[R72] Kong LB, Lekawa M, Navarro RA, McGrath J, Cohen M, Margulies DR, Hiatt JR (1996). Pedestrian-motor vehicle trauma: an analysis of injury profiles by age. J Am Coll Surg.

[R73] Konings A, Van Laer L, Van Camp G (2009). Genetic studies on noise-induced hearing loss: a review. Ear Hear.

[R74] Koshizuka S, Kanazawa K, Kobayashi N, Takazawa I, Waki Y, Shibusawa H, Shumiya S (1997). The beneficial effects of recombinant human insulin-like growth factor-I (IGF-I) on wound healing in severely wounded senescent mice. Surg Today.

[R75] Kovacs EJ, Grabowski KA, Duffner LA, Plackett TP, Gregory MS (2002). Survival and cell mediated immunity after burn injury in aged mice. J Am Aging Assoc.

[R76] Kozar RA, Arbabi S, Stein DM, Shackford SR, Barraco RD, Biffl WL, Brasel KJ, Cooper Z, Fakhry SM, Livingston D, Moore F (2015). Injury in the aged: geriatric trauma care at the crossroads. J Trauma Acute Care Surg.

[R77] Kumar A, Stoica BA, Sabirzhanov B, Burns MP, Faden AI, Loane DJ (2013). Traumatic brain injury in aged animals increases lesion size and chronically alters microglial/macrophage classical and alternative activation states. Neurobiol Aging.

[R78] Kusaka J, Koga H, Hagiwara S, Hasegawa A, Kudo K, Noguchi T (2012). Age-dependent responses to renal ischemia-reperfusion injury. J Surg Res.

[R79] Labib N, Nouh T, Winocour S, Deckelbaum D, Banici L, Fata P, Razek T, Khwaja K (2011). Severely injured geriatric population: morbidity, mortality, and risk factors. J Trauma.

[R80] Laina A, Stellos K, Stamatelopoulos K (2018). Vascular ageing: underlying mechanisms and clinical implications. Exp Gerontol.

[R81] Lamia KA, Storch K-F, Weitz CJ (2008). Physiological significance of a peripheral tissue circadian clock. Proc Natl Acad Sci U S A.

[R82] Lavie L, Weinreb O, Gershon D (1992). Age-related alterations in superoxide anion generation in mouse peritoneal macrophages studied by repeated stimulations and heat shock treatment. J Cell Physiol.

[R83] Le Prell CG (2012). Noise-induced hearing loss: from animal models to human trials. Adv Exp Med Biol.

[R84] LeBrasseur NK (2017). Physical resilience: opportunities and challenges in translation. J Gerontol A Biol Sci Med Sci.

[R85] Lee W, Moon M, Kim HG, Lee TH, Oh MS (2015). Heat stress-induced memory impairment is associated with neuroinflammation in mice. J Neuroinflammation.

[R86] Li P, Tompkins RG, Xiao W, the Inflammation and Host Response to Injury Large-Scale Collaborative Research Program (2017). KERIS: kaleidoscope of gene responses to inflammation between species. Nucleic Acids Res.

[R87] Liu F, Schafer DP, McCullough LD (2009). TTC, fluoro-Jade B and NeuN staining confirm evolving phases of infarction induced by middle cerebral artery occlusion. J Neurosci Methods.

[R88] Liu Q, Jin W-N, Liu Y, Shi K, Sun H, Zhang F, Zhang C, Gonzales RJ, Sheth KN, La Cava A, Shi F-D (2017). Brain ischemia suppresses immunity in the periphery and brain via different neurogenic innervations. Immunity.

[R89] Loh DH, Navarro J, Hagopian A, Wang LM, Deboer T, Colwell CS (2010). Rapid changes in the light/dark cycle disrupt memory of conditioned fear in mice. PLoS One.

[R90] López-Otín C, Blasco MA, Partridge L, Serrano M, Kroemer G (2013). The hallmarks of aging. Cell.

[R91] Maki T, Ihara M, Fujita Y, Nambu T, Miyashita K, Yamada M, Washida K, Nishio K, Ito H, Harada H, Yokoi H (2011). Angiogenic and vasoprotective effects of adrenomedullin on prevention of cognitive decline after chronic cerebral hypoperfusion in mice. Stroke.

[R92] Manwani B, Liu F, Scranton V, Hammond MD, Sansing LH, McCullough LD (2013). Differential effects of aging and sex on stroke induced inflammation across the lifespan. Exp Neurol.

[R93] Marquié J-C, Tucker P, Folkard S, Gentil C, Ansiau D (2015). Chronic effects of shift work on cognition: findings from the VISAT longitudinal study. Occup Environ Med.

[R94] Matsumura M, Nishioka K, Fujii T, Yoshibayashi M, Nozaki K, Nakata Y, Temma S, Ueda T, Mikawa H (1994). Age-related acute adriamycin cardiotoxicity in mice. J Mol Cell Cardiol.

[R95] Matsutani T, Kang S-C, Miyashita M, Sasajima K, Choudhry MA, Bland KI, Chaudry IH (2007). Young and middle-age associated differences in cytokeratin expression after bone fracture, tissue trauma, and hemorrhage. Am J Surg.

[R96] Matute-Bello G, Downey G, Moore BB, Groshong SD, Matthay MA, Slutsky AS, Kuebler WM, Acute Lung Injury in Animals Study Group (2011). An official American Thoracic Society workshop report: features and measurements of experimental acute lung injury in animals. Am J Respir Cell Mol Biol.

[R97] McAdams-DeMarco MA, Olorundare IO, Ying H, Warsame F, Haugen CE, Hall R, Garonzik-Wang JM, Desai NM, Walston JD, Norman SP, Segev DL (2018). Frailty and postkidney transplant health-related quality of life. Transplantation.

[R98] Mestas J, Hughes CCW (2004). Of mice and not men: differences between mouse and human immunology. J Immunol.

[R99] Miller JM, Dolan DF, Raphael Y, Altschuler RA (1998). Interactive effects of aging with noise induced hearing loss. Scand Audiol Suppl.

[R100] Mira JC, Nacionales DC, Loftus TJ, Ungaro R, Mathias B, Mohr AM, Moldawer LL, Efron PA (2018). Mouse injury model of polytrauma and shock. Methods Mol Biol.

[R101] Mistriotis P, Andreadis ST (2017). Vascular aging: molecular mechanisms and potential treatments for vascular rejuvenation. Ageing Res Rev.

[R102] Mitchell GF (2008). Effects of central arterial aging on the structure and function of the peripheral vasculature: implications for end-organ damage. J Appl Physiol.

[R103] Miura K, Goldstein RS, Morgan DG, Pasino DA, Hewitt WR, Hook JB (1987). Age-related differences in susceptibility to renal ischemia in rats. Toxicol Appl Pharmacol.

[R104] Miwa K, Tanaka M, Okazaki S, Furukado S, Yagita Y, Sakaguchi M, Mochizuki H, Kitagawa K (2014). Chronic kidney disease is associated with dementia independent of cerebral small-vessel disease. Neurology.

[R105] Miyaji T, Hu X, Yuen PST, Muramatsu Y, Iyer S, Hewitt SM, Star RA (2003). Ethyl pyruvate decreases sepsis-induced acute renal failure and multiple organ damage in aged mice. Kidney Int.

[R106] Morganti JM, Jopson TD, Liu S, Riparip L-K, Guandique CK, Gupta N, Ferguson AR, Rosi S (2015). CCR2 antagonism alters brain macrophage polarization and ameliorates cognitive dysfunction induced by traumatic brain injury. J Neurosci.

[R107] Morganti JM, Riparip L-K, Chou A, Liu S, Gupta N, Rosi S (2016). Age exacerbates the CCR2/5-mediated neuroinflammatory response to traumatic brain injury. J Neuroinflammation.

[R108] Mozaffarian D, Benjamin EJ, Go AS, Arnett DK, Blaha MJ, Cushman M, de Ferranti S, Després J-P, Fullerton HJ, Howard VJ, Huffman MD (2015). Heart disease and stroke statistics–2015 update: a report from the American Heart Association. Circulation.

[R109] Münzel T, Daiber A, Steven S, Tran LP, Ullmann E, Kossmann S, Schmidt FP, Oelze M, Xia N, Li H, Pinto A (2017). Effects of noise on vascular function, oxidative stress, and inflammation: mechanistic insight from studies in mice. Eur Heart J.

[R110] Musso CG, Jauregui JR, Macías Núñez JF (2015). Frailty phenotype and chronic kidney disease: a review of the literature. Int Urol Nephrol.

[R111] Nacionales DC, Szpila B, Ungaro R, Lopez MC, Zhang J, Gentile LF, Cuenca AL, Vanzant E, Mathias B, Jyot J, Westerveld D (2015). A detailed characterization of the dysfunctional immunity and abnormal myelopoiesis induced by severe shock and trauma in the aged. J Immunol.

[R112] Nakagawa Y, Reed L, Nakamura M, McIntosh TK, Smith DH, Saatman KE, Raghupathi R, Clemens J, Saido TC, Lee VM, Trojanowski JQ (2000). Brain trauma in aged transgenic mice induces regression of established abeta deposits. Exp Neurol.

[R113] Nguyen KD, Qiu Y, Cui X, Goh YPS, Mwangi J, David T, Mukundan L, Brombacher F, Locksley RM, Chawla A (2011). Alternatively activated macrophages produce catecholamines to sustain adaptive thermogenesis. Nature.

[R114] Nomellini V, Gomez CR, Gamelli RL, Kovacs EJ (2009). Aging and animal models of systemic insult: trauma, burn, and sepsis. Shock.

[R115] Ohlemiller KK (2006). Contributions of mouse models to understanding of age- and noise-related hearing loss. Brain Res.

[R116] Osuchowski MF, Remick DG, Lederer JA, Lang CH, Aasen AO, Aibiki M, Azevedo LC, Bahrami S, Boros M, Cooney R, Cuzzocrea S (2014). Abandon the mouse research ship? Not just yet!. Shock.

[R117] Park S-N, Back S-A, Park K-H, Seo J-H, Noh H-I, Akil O, Lustig LR, Yeo SW (2013). Comparison of functional and morphologic characteristics of mice models of noise-induced hearing loss. Auris Nasus Larynx.

[R118] Park S-Y, Marasini S, Kim G-H, Ku T, Choi C, Park M-Y, Kim E-H, Lee Y-D, Suh-Kim H, Kim S-S (2014). A method for generating a mouse model of stroke: evaluation of parameters for blood flow, behavior, and survival [corrected]. Exp Neurobiol.

[R119] Patel A, Moalem A, Cheng H, Babadjouni RM, Patel K, Hodis DM, Chandegara D, Cen S, He S, Liu Q, Mack WJ (2017). Chronic cerebral hypoperfusion induced by bilateral carotid artery stenosis causes selective recognition impairment in adult mice. Neurol Res.

[R120] Pawelczyk M, Van Laer L, Fransen E, Rajkowska E, Konings A, Carlsson P-I, Borg E, Van Camp G, Sliwinska-Kowalska M (2009). Analysis of gene polymorphisms associated with K ion circulation in the inner ear of patients susceptible and resistant to noise-induced hearing loss. Ann Hum Genet.

[R121] Pedroso FE, Spalding PB, Cheung MC, Yang R, Gutierrez JC, Bonetto A, Zhan R, Chan HL, Namias N, Koniaris LG, Zimmers TA (2012). Inflammation, organomegaly, and muscle wasting despite hyperphagia in a mouse model of burn cachexia. J Cachexia Sarcopenia Muscle.

[R122] Pfeifer R, Teuben M, Andruszkow H, Barkatali BM, Pape H-C (2016). Mortality patterns in patients with multiple trauma: a systematic review of autopsy studies. PLoS One.

[R123] Popa-Wagner A, Buga A-M, Kokaia Z (2011). Perturbed cellular response to brain injury during aging. Ageing Res Rev.

[R124] Prasher D (2009). Is there evidence that environmental noise is immunotoxic?. Noise Health.

[R125] Rademann P, Weidinger A, Drechsler S, Meszaros A, Zipperle J, Jafarmadar M, Dumitrescu S, Hacobian A, Ungelenk L, Röstel F, Kaszaki J (2017). Mitochondria-targeted antioxidants SkQ1 and MitoTEMPO failed to exert a long-term beneficial effect in murine polymicrobial Sepsis. Oxid Med Cell Longev.

[R126] Recio A, Linares C, Banegas JR, Díaz J (2016). Road traffic noise effects on cardiovascular, respiratory, and metabolic health: an integrative model of biological mechanisms. Environ Res.

[R127] Robbins NM, Swanson RA (2014). Opposing effects of glucose on stroke and reperfusion injury: acidosis, oxidative stress, and energy metabolism. Stroke.

[R128] Rosen CL, Dinapoli VA, Nagamine T, Crocco T (2005). Influence of age on stroke outcome following transient focal ischemia. J Neurosurg.

[R129] Rudolph KL, Chang S, Lee HW, Blasco M, Gottlieb GJ, Greider C, DePinho RA (1999). Longevity, stress response, and cancer in aging telomerase-deficient mice. Cell.

[R130] Saito H, Sherwood ER, Varma TK, Evers BM (2003). Effects of aging on mortality, hypothermia, and cytokine induction in mice with endotoxemia or sepsis. Mech Ageing Dev.

[R131] Salvi F, Marchetti A, D’Angelo F, Boemi M, Lattanzio F, Cherubini A (2012). Adverse drug events as a cause of hospitalization in older adults. Drug Saf.

[R132] Sanz L, Murillo-Cuesta S, Cobo P, Cediel-Algovia R, Contreras J, Rivera T, Varela-Nieto I, Avendaño C (2015). Swept-sine noise-induced damage as a hearing loss model for preclinical assays. Front Aging Neurosci.

[R133] Scherbakov N, von Haehling S, Anker SD, Dirnagl U, Doehner W (2013). Stroke induced Sarcopenia: muscle wasting and disability after stroke. Int J Cardiol.

[R134] Schneider CP, Schwacha MG, Chaudry IH (2006). Influence of gender and age on T-cell responses in a murine model of trauma-hemorrhage: differences between circulating and tissue-fixed cells. J Appl Physiol.

[R135] Schorr A, Carter C, Ladiges W (2018). The potential use of physical resilience to predict healthy aging. Pathobiol Aging Age Relat Dis.

[R136] Schosserer M, Grillari J, Wolfrum C, Scheideler M (2018). Age-induced changes in white, brite, and brown adipose depots: a mini-review. Gerontology.

[R137] Schwalm MT, Pasquali M, Miguel SP, Dos Santos JPA, Vuolo F, Comim CM, Petronilho F, Quevedo J, Gelain DP, Moreira JCF, Ritter C (2014). Acute brain inflammation and oxidative damage are related to long-term cognitive deficits and markers of neurodegeneration in sepsis-survivor rats. Mol Neurobiol.

[R138] Secor D, Li F, Ellis CG, Sharpe MD, Gross PL, Wilson JX, Tyml K (2010). Impaired microvascular perfusion in sepsis requires activated coagulation and P-selectin-mediated platelet adhesion in capillaries. Intensive Care Med.

[R139] Sessler DI (2001). Complications and treatment of mild hypothermia. Anesthesiology.

[R140] Shahidi B, Shah SB, Esparza M, Head BP, Ward SR (2018). Skeletal muscle atrophy and degeneration in a mouse model of traumatic brain injury. J Neurotrauma.

[R141] Shallo H, Plackett TP, Heinrich SA, Kovacs EJ (2003). Monocyte chemoattractant protein-1 (MCP-1) and macrophage infiltration into the skin after burn injury in aged mice. Burns.

[R142] Shay T, Jojic V, Zuk O, Rothamel K, Puyraimond-Zemmour D, Feng T, Wakamatsu E, Benoist C, Koller D, Regev A (2013). ImmGen Consortium: Conservation and divergence in the transcriptional programs of the human and mouse immune systems. Proc Natl Acad Sci U S A.

[R143] Shefer VI, Talan MI, Engel BT (1996). Heat loss during cold exposure in adult and aged C57BL/6J mice. Exp Gerontol.

[R144] Shen Z, Ruan Q, Yu Z, Sun Z (2017). Chronic kidney disease-related physical frailty and cognitive impairment: a systemic review. Geriatr Gerontol Int.

[R145] Shibata M, Ohtani R, Ihara M, Tomimoto H (2004). White matter lesions and glial activation in a novel mouse model of chronic cerebral hypoperfusion. Stroke.

[R146] Shim R, Wong CHY (2016). Ischemia, immunosuppression and infection–tackling the predicaments of post-stroke complications. Int J Mol Sci.

[R147] Shrum B, Anantha RV, Xu SX, Donnelly M, Haeryfar SMM, McCormick JK, Mele T (2014). A robust scoring system to evaluate sepsis severity in an animal model. BMC Res Notes.

[R148] Sikandaner HE, Park SY, Kim MJ, Park SN, Yang DW (2017). Neuroprotective effects of sildenafil against oxidative stress and memory dysfunction in mice exposed to noise stress. Behav Brain Res.

[R149] Skorupski AM, Zhang J, Ferguson D, Lawrence F, Hankenson FC (2017). Quantification of induced hypothermia from aseptic scrub applications during rodent surgery preparation. J Am Assoc Lab Anim Sci.

[R150] Sliwinska-Kowalska M, Davis A (2012). Noise-induced hearing loss. Noise Health.

[R151] Springer J, Schust S, Peske K, Tschirner A, Rex A, Engel O, Scherbakov N, Meisel A, von Haehling S, Boschmann M, Anker SD (2014). Catabolic signaling and muscle wasting after acute ischemic stroke in mice: indication for a stroke-specific sarcopenia. Stroke.

[R152] Starr ME, Steele AM, Cohen DA, Saito H (2016). Short-term dietary restriction rescues mice from lethal abdominal Sepsis and endotoxemia and reduces the inflammatory/coagulant potential of adipose tissue. Crit Care Med.

[R153] Storer JB (1962). Evaluation of radiation response as an index of aging in mice. Radiat Res.

[R154] Sumbria RK, Grigoryan MM, Vasilevko V, Paganini-Hill A, Kilday K, Kim R, Cribbs DH, Fisher MJ (2018). Aging exacerbates development of cerebral microbleeds in a mouse model. J Neuroinflammation.

[R155] Swift ME, Burns AL, Gray KL, DiPietro LA (2001). Age-related alterations in the inflammatory response to dermal injury. J Invest Dermatol.

[R156] Swoap SJ, Li C, Wess J, Parsons AD, Williams TD, Overton JM (2008). Vagal tone dominates autonomic control of mouse heart rate at thermoneutrality. Am J Physiol Heart Circ Physiol.

[R157] Tachibana M, Ago T, Wakisaka Y, Kuroda J, Shijo M, Yoshikawa Y, Komori M, Nishimura A, Makihara N, Nakamura K, Kitazono T (2017). Early reperfusion after brain ischemia has beneficial effects beyond rescuing neurons. Stroke.

[R158] Talan M, Engel BT (1984). An improved cold stress test for aging mice. Exp Gerontol.

[R159] Talan MI, Engel BT, Whitaker JR (1985). A longitudinal study of tolerance to cold stress among C57BL/6J mice. J Gerontol.

[R160] Tateda K, Matsumoto T, Miyazaki S, Yamaguchi K (1996). Lipopolysaccharide-induced lethality and cytokine production in aged mice. Infect Immun.

[R161] Tatelman HM, Talan MI (1990). Metabolic heat production during repeated cold stress in adult and aged male C57BL/6J mice. J Gerontol.

[R162] Timmermans K, Kox M, Scheffer GJ, Pickkers P (2016). Danger in the Intensive Care Unit: DAMPS in critically ill patients. Shock.

[R163] Tobías A, Recio A, Díaz J, Linares C (2015). Health impact assessment of traffic noise in Madrid (Spain). Environ Res.

[R164] Tseng L-S, Chen S-H, Lin M-T, Lin Y-C (2014). Umbilical cord blood-derived stem cells improve heat tolerance and hypothalamic damage in heat stressed mice. Biomed Res Int.

[R165] Tyml K, Swarbreck S, Pape C, Secor D, Koropatnick J, Feng Q, Veldhuizen RAW, Gill SE (2017). Voluntary running exercise protects against sepsis-induced early inflammatory and pro-coagulant responses in aged mice. Crit Care.

[R166] Vallanat B, Anderson SP, Brown-Borg HM, Ren H, Kersten S, Jonnalagadda S, Srinivasan R, Corton JC (2010). Analysis of the heat shock response in mouse liver reveals transcriptional dependence on the nuclear receptor peroxisome proliferator-activated receptor alpha (PPARalpha). BMC Genomics.

[R167] van Gool WA, van de Beek D, Eikelenboom P (2010). Systemic infection and delirium: when cytokines and acetylcholine collide. Lancet (London, England).

[R168] Vatner DE, Zhang J, Oydanich M, Guers J, Katsyuba E, Yan L, Sinclair D, Auwerx J, Vatner SF (2018). Enhanced longevity and metabolism by brown adipose tissue with disruption of the regulator of G protein signaling 14. Aging Cell.

[R169] Vaughan MS, Vaughan RW, Cork RC (1981). Postoperative hypothermia in adults: relationship of age, anesthesia, and shivering to rewarming. Anesth Analg.

[R170] Veilleux-Lemieux D, Castel A, Carrier D, Beaudry F, Vachon P (2013). Pharmacokinetics of ketamine and xylazine in young and old Sprague-Dawley rats. J Am Assoc Lab Anim Sci.

[R171] Walker SR, Wagner M, Tangri N (2014). Chronic kidney disease, frailty, and unsuccessful aging: a review. J Ren Nutr.

[R172] Wang XH, Mitch WE (2013). Muscle wasting from kidney failure-a model for catabolic conditions. Int J Biochem Cell Biol.

[R173] Wei Q, Dong Z (2012). Mouse model of ischemic acute kidney injury: technical notes and tricks. Am J Physiol Ren Physiol.

[R174] Wen J, Zeng M, Shu Y, Guo D, Sun Y, Guo Z, Wang Y, Liu Z, Zhou H, Zhang W (2015). Aging increases the susceptibility of cisplatin-induced nephrotoxicity. Age (Dordr).

[R175] Whitehead JC, Hildebrand BA, Sun M, Rockwood MR, Rose RA, Rockwood K, Howlett SE (2014). A clinical frailty index in aging mice: comparisons with frailty index data in humans. J Gerontol A Biol Sci Med Sci.

[R176] Whitman S, Good G, Donoghue ER, Benbow N, Shou W, Mou S (1997). Mortality in Chicago attributed to the July 1995 heat wave. Am J Public Health.

[R177] Whitson HE, Duan-Porter W, Schmader KE, Morey MC, Cohen HJ, Colón-Emeric CS (2016). Physical resilience in older adults: systematic review and development of an emerging construct. J Gerontol A Biol Sci Med Sci.

[R178] Wolf G, Lotan A, Lifschytz T, Ben-Ari H, Kreisel Merzel T, Tatarskyy P, Valitzky M, Mernick B, Avidan E, Koroukhov N, Lerer B (2017). Differentially severe cognitive effects of compromised cerebral blood flow in aged mice: association with myelin degradation and microglia activation. Front Aging Neurosci.

[R179] Wu J, Zhang M, Hao S, Jia M, Ji M, Qiu L, Sun X, Yang J, Li K (2015). Mitochondria-targeted peptide reverses mitochondrial dysfunction and cognitive deficits in sepsis-associated encephalopathy. Mol Neurobiol.

[R180] Wyse CA, Selman C, Page MM, Coogan AN, Hazlerigg DG (2011). Circadian desynchrony and metabolic dysfunction; did light pollution make us fat?. Med Hypotheses.

[R181] Xu X, Fan M, He X, Liu J, Qin J, Ye J (2014). Aging aggravates long-term renal ischemia-reperfusion injury in a rat model. J Surg Res.

[R182] Yamazaki S, Numano R, Abe M, Hida A, Takahashi R, Ueda M, Block GD, Sakaki Y, Menaker M, Tei H (2000). Resetting central and peripheral circadian oscillators in transgenic rats. Science.

[R183] Zhou X, Qi Y, Chen T (2017). Long-term pre-treatment of antioxidant Ginkgo biloba extract EGb-761 attenuates cerebral-ischemia-induced neuronal damage in aged mice. Biomed Pharmacother.

